# Infrared sensitive mixed phase of V_7_O_16_ and V_2_O_5_ thin-films†

**DOI:** 10.1039/d3ra00752a

**Published:** 2023-05-22

**Authors:** Anchal Rana, Aditya Yadav, Govind Gupta, Abhimanyu Rana

**Affiliations:** a Centre for Advanced Materials and Devices, School of Engineering and Technology, BML Munjal University Sidhrawali Gurugram-122413 Haryana India rana.abhimanyu@gmail.com; b CSIR-National Physical Laboratory K. S. Krishnan Marg New Delhi 110012 India

## Abstract

We report an infrared (IR) sensitive mixed phase of V_7_O_16_ and V_2_O_5_ thin films, grown by cathodic vacuum arc-deposition on glass substrates at relatively low temperatures. We have found that the mixed phase of V_7_O_16_ and V_2_O_5_ can be stabilized by post-annealing of amorphous V_*x*_O_*y*_ between 300–400 °C, which gets fully converted into V_2_O_5_ after annealing at higher temperatures ∼450 °C. The local conversion from V_*x*_O_*y*_ to V_2_O_5_ has also been demonstrated by applying different laser powers in Raman spectroscopy measurements. The optical transmission of these films increases as the content of V_2_O_5_ increases but the electrical conductivity and the optical bandgap decrease. These results are explained by the role of defects (oxygen vacancies) through the photoluminescence (PL) and time-resolved photoluminescence (TRPL) measurements. The IR sensitivity of the mixed phase is explained by the plasmonic absorption by the V_7_O_16_ degenerate semiconductor.

## Abstract 2

Mixed phase of V_7_O_16_ and V_2_O_5_ thin films for IR sensing and smart windows.

## Graphical abstract

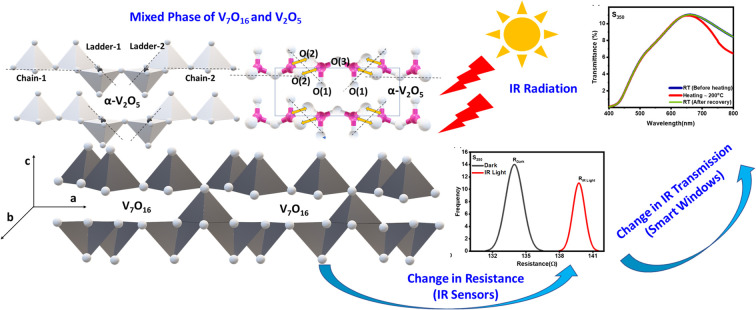

## Introduction

Infrared (IR) sensitive materials have been of significant interest for IR sensors and smart windows in which the resistance or the optical transmission can be modulated by heating or IR radiation.^1,2^ Therefore, many low-bandgap semiconductors,^3,4^ quantum-well heterostructures^5^ and high-temperature-superconductors^6,7^ are intensively studied. However, these materials need to be cooled at lower temperatures for these applications. Vanadium-based oxides, found in more than 52 stable and metastable phases, have been investigated for developing uncooled devices as they show metal–insulator transitions (MIT) close to room temperatures under different external stimuli such as thermal, electrical, and optical.^8–14^ Vanadium (V) can have oxidation states varying from +2 to +5, where V_2_O_3_, VO_2_, and V_2_O_5_ are the most studied phases but other non-stoichiometric phases such as V_3_O_7_, V_4_O_9_, V_6_O_13_, V_7_O_16_ have also been reported, that occur due to the presence of more than one valency of V atom in a single crystal structure.^14–17^ Although, VO_2_ (having V^4+^ valency) has been the most preferred phase for IR sensors^10,11,18^ and smart windows due to sharp MIT close to room temperature,^19–22^ it has limitations of growing in the right phase on arbitrary substrates at low temperatures. On the other hand, V_2_O_5_ having a layered structure is important for energy storage electrodes,^23–26^ gas sensors^27–29^ and chromogenic applications.^30–32^ However, the applicability of the pure V_2_O_5_ phase is again largely restricted due to high temperature growth and poor electrical conductivity. The mixed phase of VO_2_ and V_2_O_5_ has been reported to give enhanced IR sensitivity.^33^ Other phases such as V_7_O_16_ could be promising for these applications which has the mixture of both V^4+^ and V^5+^ arranged in a layered crystal structure.^15,34,35^ However, there are very few reports on V_7_O_16_ phase which has been reported during chemical synthesis of V_2_O_5_ nanotubes^28,35^ or in thin film form when grown under oxygen deficient conditions using pulsed laser deposition^29^ and atomic layer deposition.^15^ There is a clear research gap for in-depth understanding of electronic and optical properties of V_7_O_16_ for any practical application in smart windows and IR sensors. Here, we systematically tune the contents of V_7_O_16_ and V_2_O_5_ in a mixed phase (also changing the V^4+^ and V^5+^ ratio) by post-annealing of amorphous vanadium oxide thin films grown by cathodic vacuum arc-deposition. We report a strong modulation of electrical resistance and optical transmission in these films with IR radiation and temperature.

## Materials and methods

First an amorphous vanadium oxide thin films were grown on high-grade barium-borosilicate 7059 glass substrates using cathodic vacuum arc-deposition by evaporating a vanadium metal target (∼99.99% purity) in the presence of oxygen at a partial pressure of ∼1 × 10^−3^ mbar, arc current of ∼150 A, substrate temperature ∼200 °C and substrate bias of −60 V. These films were then taken out and annealed in air at different temperatures *i.e.*, ∼300 °C, ∼350 °C, ∼400 °C, ∼450 °C, and 520 °C for 2 hours. The unannealed sample will be called as S_as-grown_ and other annealed sampled as S_300_, S_350_, S_400_, S_450_, and S_520_ in this manuscript. The Raman spectroscopy was carried out using a commercial spectrometer by WiTech (Alpha 300) having a green laser of 532 nm wavelength. The crystal structure was confirmed by X-ray diffraction (XRD, Panalytical Empyrean Model) by scanning 2*θ* ranging from 10° to 90° at a step size of 0.02° using Cu Kα radiation (*λ* ∼ 1.54 Å). The X-ray photoelectron spectroscopy (XPS) was carried out using XPS/ESCA, K-ALPHA+, Thermo Fisher Scientific. The deconvolution and fitting of XPS data of was performed using XPSpeak41 software. The optical transmission spectra were obtained in the wavelength range from 380 nm to 800 nm using a UV-visible spectrometer by PerkinElmer LAMBDA 365 model. Electrical measurements were performed using a four-probe setup equipped with Keithley electrometers. The IR sensing measurements were carried out using the IR source by Newport model 6363IR having intensity ∼438 W m^−2^. Photoluminescence (PL) and time-resolved photoluminescence (TRPL) were recorded at the excitation wavelength of ∼350 nm and ∼266 nm (pulsed) using Edinburgh (FLS 980D2D2) set-up.

## Results and discussion

**Fig. 1:**
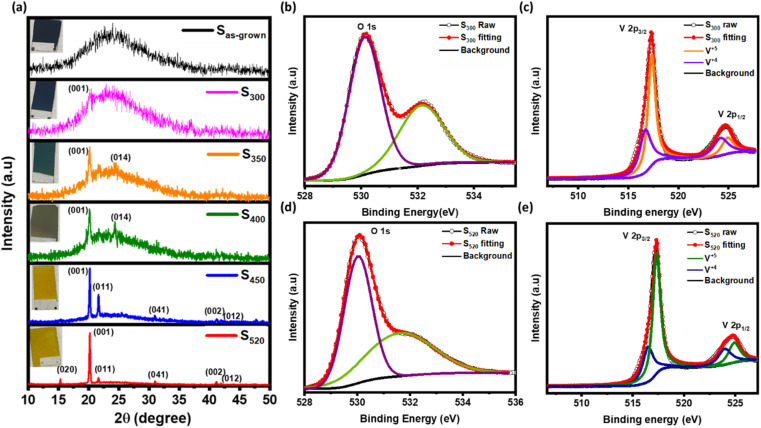
(a) X-ray diffraction (XRD) spectra of vanadium oxide thin films annealed at various temperature. X-ray photoelectron spectroscopy (XPS) spectra of (b and c) S_300_ and (d and e) S_520_ samples shows V(2p) and O(1s) peaks.

The XRD patterns of S_as-grown_, S_300_, S_350_, S_400_, S_450_, and S_520_ are shown in Fig. 1(a) with their corresponding photographs in the inset. Apparently, the photographic images show a clear change in colour from black (of amorphous films) to the yellowish orange after annealing. There is also a clear transition in XRD spectra from a broad hump in S_as-grown_ to sharp peaks in annealed samples at ∼15.3°, ∼20.18°, ∼21.65°, ∼31.04°, ∼41.21° and ∼41.87° related to (020), (001), (011), (040), (002), and (012) crystalline planes of the pure orthorhombic phase of V_2_O_5_ respectively. However, the peak at ∼24.5° confirms the presence of the V_7_O_16_ triclinic phase^15,34^ in S_350_ and S_400_ samples.

To further confirm the presence of V_7_O_16_, the X-ray photoelectron spectroscopy (XPS) were performed on S_300_ and S_520_, as shown in Fig. 1(b, c) and (d, e) respectively. The binding energy peak ∼284.8 eV of carbon 1s orbital was used to compensate the shifts in other peaks due to charging. The XPS spectra for 2p orbitals of vanadium (V) has two peaks corresponding to 2p_3/2_ and 2p_1/2_ due to the spin–orbit splitting of ∼7.5 eV. However, each peak 2p_3/2_ and 2p_1/2_ were further deconvoluted that correspond to the presence of V^4+^ (∼516.5 eV & ∼524 eV) and V^5+^ (∼517.5 eV & ∼525 eV).^15,36^ The peak at ∼530 eV is related to 1s oxygen in vanadium oxygen bonds,^37,38^ but other peaks at higher energies ∼532 eV are due to free hydroxyl groups adsorbed on the hydrophilic surfaces.^39^ The ratio of V^4+^ and V^5+^ contents in S_300_ and S_520_, estimated by the dividing the area under the curve of respective peaks, is found to be consistent with literature,^15,40^ implying higher V^4+^ content in these samples having V_7_O_16_ phase. In S_520_, peaks corresponding to V^4+^ were observed because of the photoreduction of V^5+^ in V_2_O_5_ phase.^41^

**Fig. 2:**
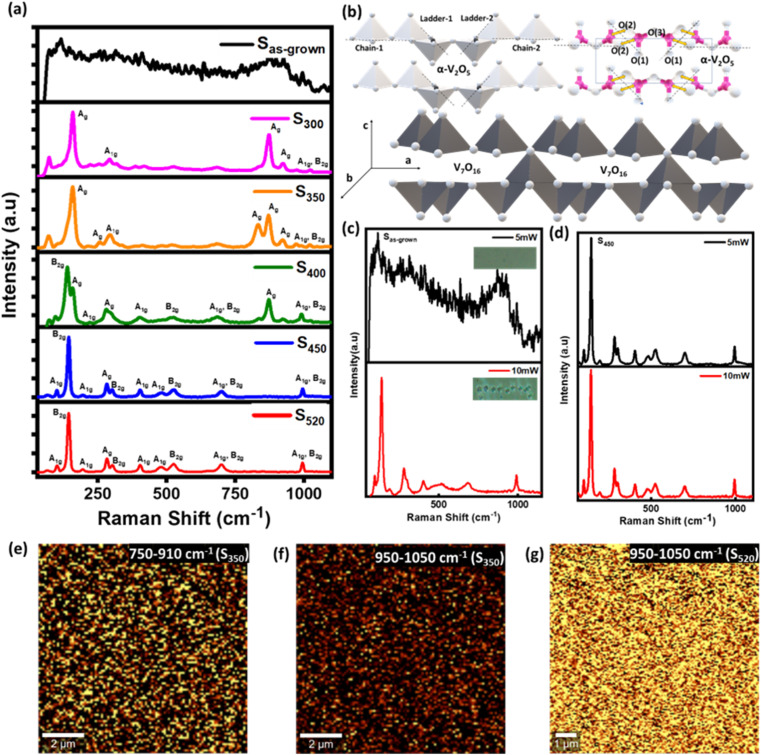
(a) Raman spectra of vanadium oxide thin films annealed at various temperatures; (b) the crystal structure of α-V_2_O_5_ and V_7_O_16_; (c and d) S_as-grown_ and S_450_ samples at the laser power 5 mW and 10 mW laser power; (e–g) Raman mapping of the S_350_ and S_520_ samples.

The Raman spectroscopic results of these films are compared in Fig. 2. Due to the amorphous nature of S_as-grown_ film, no sharp peaks were observed but as annealing temperature exceeds 300 °C, sharp peaks start appearing. The Raman spectra for S_450_ and S_520_ shows peaks at ∼101, ∼144, ∼194, ∼282, ∼304, ∼404, ∼483, ∼507, ∼528, ∼699, and ∼992 cm^−1^ due to different vibrational modes of V_2_O_5_ phase,^14,15,42–44^ as description given in Table 1. Especially, the strongest peak at ∼144 cm^−1^ arises from the V–O–V chains of the layered structure of α-V_2_O_5_, as shown in Fig. 2(b). For the samples annealed below 400 °C (S_300_ and S_350_), the peaks around ∼158, ∼255, ∼294, ∼832, ∼870, ∼970 cm^−1^ confirms the presence of V_7_O_16_ phase, also consistent with the earlier studies.^14,44,45^ It is to be noted that the presence of other polymorphs of V_2_O_5_ (β- γ-)^14,26,42,44^ cannot be completely ruled out using Raman spectroscopy due to overlapping peak positions.^14^Fig. 2(b) shows the schematic of V_7_O_16_ phase where the layered structure resembles to α-V_2_O_5_, causing the peak ∼144 cm^−1^ to be shifted to higher wavenumber ∼158 cm^−1^. However, the other vibrational modes related to ladder steps (LS) seems to be missing in V_7_O_16_, indicating the absence of LS in this phase. Clearly, other peaks at ∼255, ∼294, ∼832, ∼870, and ∼970 cm^−1^ could be assigned to the vibrational modes of V=O bonds (A_g_ symmetry).^15^ Also, the Raman spectroscopy performed at different laser powers (5 mW to 10 mW) in Fig. 2(c) and (d) shows the immediate conversion of amorphous phase to V_2_O_5_, unlike the annealed samples. Similar conversion was also observed by other groups in case of chemically synthesized VO_*x*_ nanotubes.^43^ This study opens further questions of stabilizing the intermediate phase of V_7_O_16_ using laser heating and open the opportunities for applications in laser writing for high security tags and detection Fig. 2(e)–(g) shows the Raman mapping of S_350_ and S_520_ samples. Since the Raman spectra of the S_350_ show the characteristic peaks at ∼832 cm^−1^, 870 cm^−1^, 920 cm^−1^ and S_520_ at 994 cm^−1^, we performed Raman mapping by selecting two wavelengths range from 750–910 cm^−1^ and 950–1050 cm^−1^ so that we could capture the regions of V_7_O_16_ and V_2_O_5_ spatially in 10 × 10 μm^2^ area. In Fig. 2(e) and (f), for S_350_ both V_7_O_16_ and V_2_O_5_ phases can be seen. On the other hand, for S_520_ the brighter region mostly cover the V_2_O_5_ with some minor black regions due to some other phases.

**Table 1:** Raman-active phonon modes in mixed phase of V_7_O_16_ and V_2_O_5_ thin-films

*ν* (cm^−1^)	Description	Phonon modes
101	In phase rotation of the V_2_O_4_ ladder step (LS) unit in *b*-axis (crystalline V_2_O_5_)^14,42^	A_1g_
144	Skeleton bending of V–O–V chain (crystalline V_2_O_5_)^14^	B_2g_
158	Skeleton bending of V–O–V chain (crystalline V_7_O_16_)^14,15^	A_g_
194	Bending of O(2)–V–O(3) (crystalline V_2_O_5_)^14^	A_1g_
255	Vibration of the V=O bond (crystalline V_7_O_16_)	A_g_
282	Bending of V=O (crystalline V_2_O_5_)^14^	A_1g_
294	Bending of V=O (crystalline V_7_O_16_)	A_g_
304	Bending of V–O(2) (crystalline V_2_O_5_)^14^	B_2g_
404	Angle-bending of V–O(3)–V bonds (crystalline V_2_O_5_)^14^	A_1g_
483	Symmetric stretching of V–O(3)–V bonds (crystalline V_2_O_5_)^14^	A_1g_
507	Stretching vibrations of V–O(2) bonds (crystalline V_2_O_5_)^14^	B_2g_
528	Stretching vibrations of V–O(2) bonds (crystalline V_2_O_5_)^14^	A_1g_
699	Asymmetric stretching of V–O(2)–V bridge (crystalline V_2_O_5_)	A_1g_, B_2g_
832	Stretching vibration of the V=O bond (crystalline V_7_O_16_)^14,15^	A_g_
870	Stretching vibration of the V=O bond (crystalline V_7_O_16_)^14,15^	A_g_
920	Stretching vibrations of V^4+^=O bond (due to oxygen vacancies)^15^	A_g_
992	Stretching vibration of V=O (crystalline V_2_O_5_)^14^	A_1g_, B_2g_

**Fig. 3:**
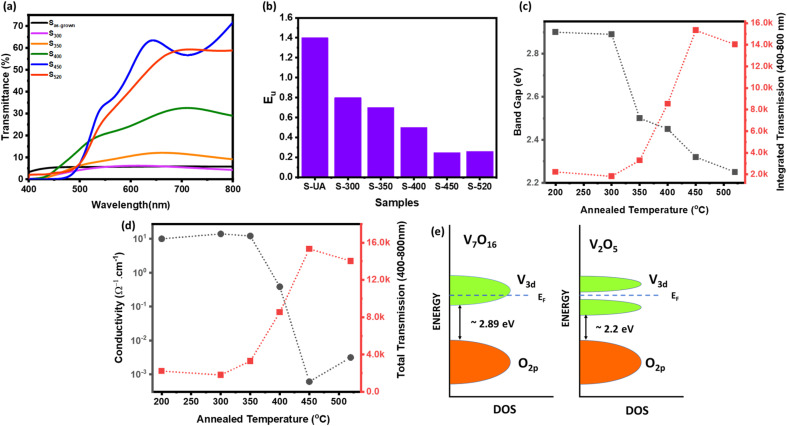
(a) Optical transmission of vanadium oxide thin films annealed at different temperatures (b) Urbach energy of the samples (c) correlated with bandgaps and (d) conductivity of thin films (e) schematic band diagram of V_2_O_5_ and V_7_O_16_.

Fig. 3(a) shows the optical transmission spectra of S_as-grown_, S_300_, S_350_, S_400_, S_450_, and S_520_. Apparently, the transmittance gradually increases with increasing the annealing temperature, and the maximum transmittance was observed in sample S_400_. The optical band gap was calculated for all the samples by using Tauc's equations.^46^ The calculated energy band gap of these samples decreases from ∼2.85 eV to 2.2 eV for the samples annealed at higher temperatures [Fig. 3(c)].

Usually, the amorphous semiconductors exhibit band-tailing due to the structural disorder which can be quantified as Urbach energy (*E*_u_) shown in Fig. 3(b). The amorphous S_as-grown_ sample clearly having more defects shows highest *E*_u_ value which decreases by increasing annealing temperature, that indicate the defects are significantly reduced after annealing. The electrical conductivity (measured by four-probe) also decreases for the samples annealed at higher temperatures, as shown in Fig. 3(d). Fig. 3(c) and (d) also shows the optical transmission integrated over the wavelength from 400–800 nm plotted on right *Y*-axis, having clear correlation with the electronic properties. The results are explained by considering a band-model shown in Fig. 3(e). Although the band gap of V_7_O_16_ is higher than the V_2_O_5_, it still has high conductivity due to the presence of more V^4+^ content, that contribute extra electrons in 3d conduction band. Also, the amorphous and low-temperature annealed samples are expected to have large number of defects and oxygen vacancies, that can even push the Fermi level into the conduction band, making V_7_O_16_ a degenerate semiconductor.^23^ Therefore, the overall conductivity of mixed phase is higher than the V_2_O_5_ due to the presence of V_7_O_16_. On the other hand, the samples annealed at high-temperature are defects free, despite having a lower bandgap and the Fermi level lies in the band gap. The dynamic resistance *versus* temperature measurements also confirms the metallic behavior of mixed phase, where the resistance is found to increase with temperature compared to pure V_2_O_5_ showing a typical decrease in resistance with temperature of semiconducting behavior, as shown in ESI Fig. S1.† The correlation of transmission with conductivity in Fig. 3(c) and (d) can be explained by reflection and absorption caused by higher metallicity and defect states, respectively.

**Fig. 4:**
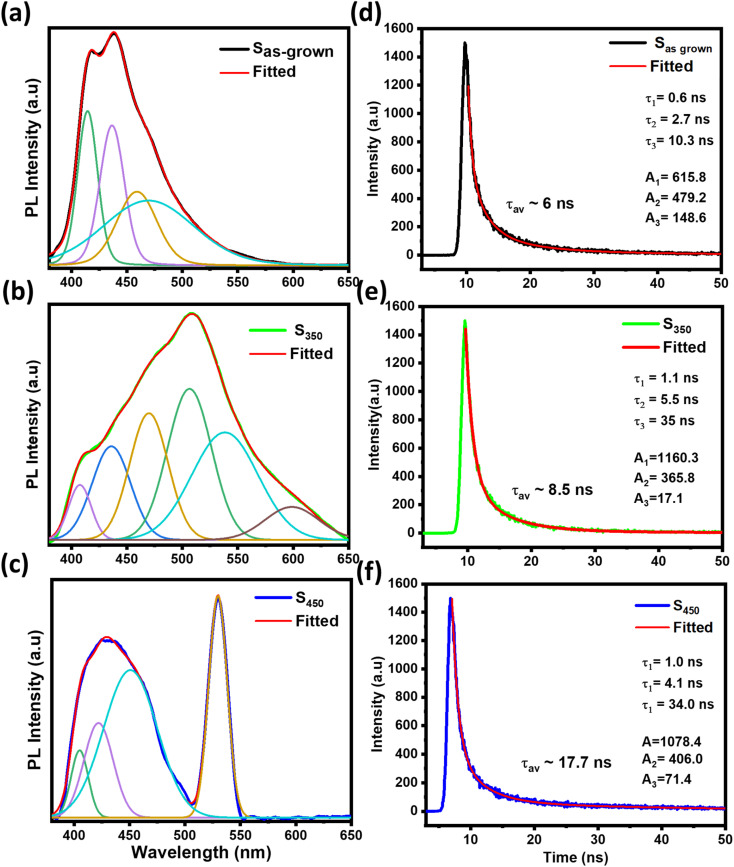
(a–c) The photoluminescence (PL) and (d–f) time-resolved photoluminescence (TRPL) spectra of S_as-grown_, S_350_ and S_450_ samples.

The photoluminescence (PL) and time-resolved-photoluminescence (TRPL) results of S_as-grown_, S_350_, and S_450_ are shown in Fig. 4. The peaks in PL spectra were deconvoluted using Gaussian function. The highest intensity peaks at ∼438 nm, ∼507 nm, ∼530 nm can be assigned to the band-edge transitions from V_3d_ conduction band to O_2p_ valence band,^37^ matching the bandgap value of each sample measured by UV-vis measurement in Fig. 3. The peaks at longer wavelengths in NIR can be assigned to the electronic transitions in mid-gap states due to oxygen vacancies, and the peaks at shorter wavelengths in UV are due to the transitions from higher V_3d_ states to O_2p_ bands.^37^ Apparently, the more peaks in NIR are observed in amorphous and mixed phase samples due to large number of defects and oxygen vacancies compared to V_2_O_5_. The increased bandgap of mixed phase can be caused by the Burstein–Moss shift in PL spectra as a result of degenerate semiconducting behaviour of V_7_O_16_.^23,47^

To further investigate the nature of defects, we have measured the carrier life using TRPL as shown in Fig. 4(b) of S_as-grown_, S_350_, and S_450_. In TRPL, the peak intensity decay (*I*_TRPL_) with time (*t*) can be fitted using the following equation
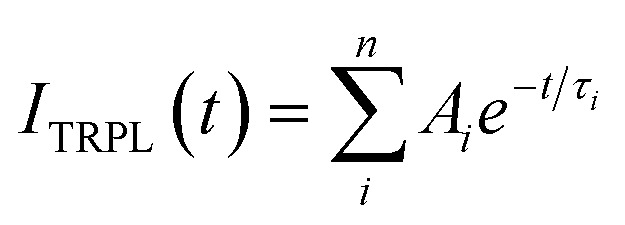
where *A*_*i*_, *τ*_*i*_ are constants representing the amplitude and the lifetime of carriers. Here, we have used component *i* = 3 to fit the experimental curve and calculated the average decay time using the following expression
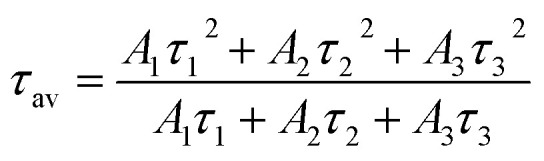


The extracted fitting parameters and average decay time are shown in Table 2. The decay constants *τ*_1_ and *τ*_2_ attributes to the fast decay through trap-mediated recombination, while *τ*_3_ corresponds to slow decay through radiative recombination.^37^ The average decay time of S_450_ is higher compared than other two samples due to the reduction in oxygen vacancies in the samples, which agrees well with the previous observations.^48^ These results are in good agreement with the Urbach energy measurements shown in Fig. 3(b).

**Table 2:** Time-resolved photoluminescence (TRPL) parameters for the S_as-grown_, S_350_ and S_450_ samples

Sample	*A*_1_	*A*_2_	*A*_3_	*τ*_1_ (ns)	*τ*_2_ (ns)	*τ*_3_ (ns)	*τ*_average_ (ns)
S_as-grown_	615.8	479.2	148.6	0.6	2.7	10.3	6.1
S_350_	1160.3	365.7	17.1	1.1	5.5	35.0	8.5
S_450_	1078.4	406.0	71.4	1.0	4.1	33.9	17.7

**Fig. 5:**
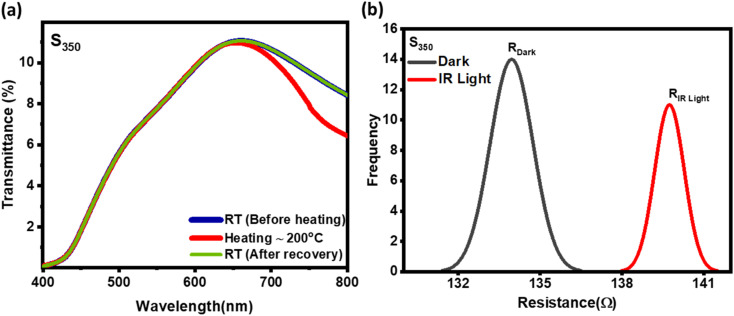
(a) UV-spectra of the mixed phase before and after heating at 200 °C (b) the distribution of resistance value before and after illumination of under IR radiation.

Finally, we demonstrate the sensitivity of our mixed phase sample by heating and IR radiation as shown in Fig. 5(a) and (b) respectively. Fig. 5(a) illustrate the optical transmission taken before and after heating the sample at 200 °C outside and then transferred to the UV-vis spectroscopy. Interestingly, a clear change in the transmission was only observed for the wavelengths higher than ∼600 nm in near-IR (NIR) region. Indeed, no major change in the spectrum was observed in the visible range, indicating that these films are more sensitive to IR. Clearly, there will be some variations in temperature while taking the optical measurements, but the overall spectrum is always fully recovered as the samples is cooled back to the room temperature naturally.

Also, V_7_O_16_ being a degenerate semiconductor could also be suitable candidate for NIR plasmonics. Indeed, the broad peak ∼650 nm in the UV-vis spectrum could be due to the NIR plasmons absorption.^23^ Since the plasmonic frequency 
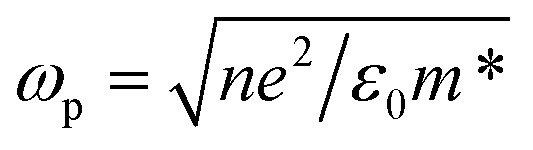
 is related to the carrier concentration (*n*) and the effective mass (m*), where *e* and *ε*_0_ are electronic charge and the permittivity of free space, respectively. The measured value of resonant frequency ∼4.6 × 10^14^ Hz gives the carrier concentration ∼10^20^ to 10^21^ cm^−3^, which is also consistent with our conductivity values considering the theoretical values of mobilities and effective mass of vanadium oxides (1–1.7*m*_e_).^49,50^ The IR sensitivity in optical transmission is due to the plasmonic absorption and consistent with the earlier studied on the mixed phase of metallic VO_2_ and insulating V_2_O_5_.^33^ Also, the increase in resistance under IR radiation seen in Fig. 5(b) could be due to the electron–electron and electron–phonon scatterings as observed in metals and degenerate semiconductors in the resistance *versus* temperature measurements. Clearly, more low temperature transport measurements are required to further investigate the transport properties, but these results are encouraging for their promising use in smart-windows and IR sensors.

## Conclusions

In conclusion, we provide a detailed in-depth understanding of electronic and optical properties of V_7_O_16_ and systematically tune the electronic and optical properties of a mixed phase by changing the V^4+^ and V^5+^ contents. We report a strong modulation in the conductivity and optical transmission with infrared radiation and temperature. These results are very promising for vanadium oxide based uncooled-IR sensors and smart windows that can be grown at low temperatures using a commercially viable cathodic vacuum arc-deposition technique.

## Conflicts of interest

There are no conflicts to declare.

## Supplementary Material

RA-013-D3RA00752A-s001
