# Correction: Infrared sensitive mixed phase of V_7_O_16_ and V_2_O_5_ thin-films

**DOI:** 10.1039/d3ra90051g

**Published:** 2023-06-13

**Authors:** Anchal Rana, Aditya Yadav, Govind Gupta, Abhimanyu Rana

**Affiliations:** a Centre for Advanced Materials and Devices, School of Engineering and Technology, BML Munjal University Sidhrawali Gurugram 122413 Haryana India rana.abhimanyu@gmail.com; b CSIR-National Physical Laboratory K. S. Krishnan Marg New Delhi 110012 India

## Abstract

Correction for ‘Infrared sensitive mixed phase of V_7_O_16_ and V_2_O_5_ thin-films’ by Anchal Rana *et al.*, *RSC Adv.*, 2023, **13**, 15334–15341, https://doi.org/10.1039/D3RA00752A.

The authors regret that incorrect details were given for ref. 22 in the original article. The correct version of ref. 22 is given below as ref. 1.

The Royal Society of Chemistry apologises for these errors and any consequent inconvenience to authors and readers.

## Supplementary Material
