# Supplementary material for: Infrared sensitive mixed phase of V7O16 and V2O5 thin-films
Source: RSC Adv. 2023 May 22;13(22):15334–41. doi: 10.1039/d3ra00752a (PMC10201198; doi:10.1039/d3ra00752a)
Supplement: RA-013-D3RA00752A-s001 [file RA-013-D3RA00752A-s001.pdf]

Electronic Supplementary Material (ESI) for RSC Advances.
This journal is © The Royal Society of Chemistry 2023

Supplementary Information

Infrared Sensitive Mixed Phase of V_7O_{16} and V_2O_5 Thin-Films

Anchal Rana[1], Aditya Yadav[2], Govind Gupta[2], Abhimanyu Rana[1*]

[1]Centre for Advanced Materials and Devices, School of Engineering and Technology, BML Munjal University, Sidhrawali, Gurugram-122413, Haryana, India

[2]CSIR-National Physical Laboratory, K.S. Krishnan Marg, New Delhi, 110012, India

*rana.abhimanyu@gmail.com

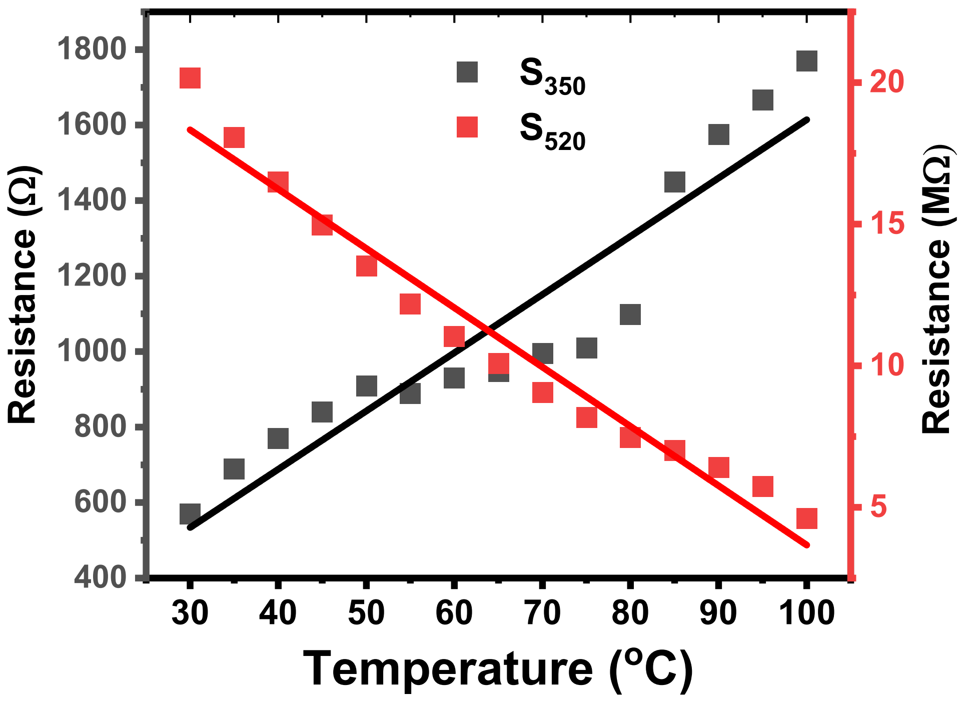

Figure S1: Resistance versus temperature curve for mixed phase and V_2O_5

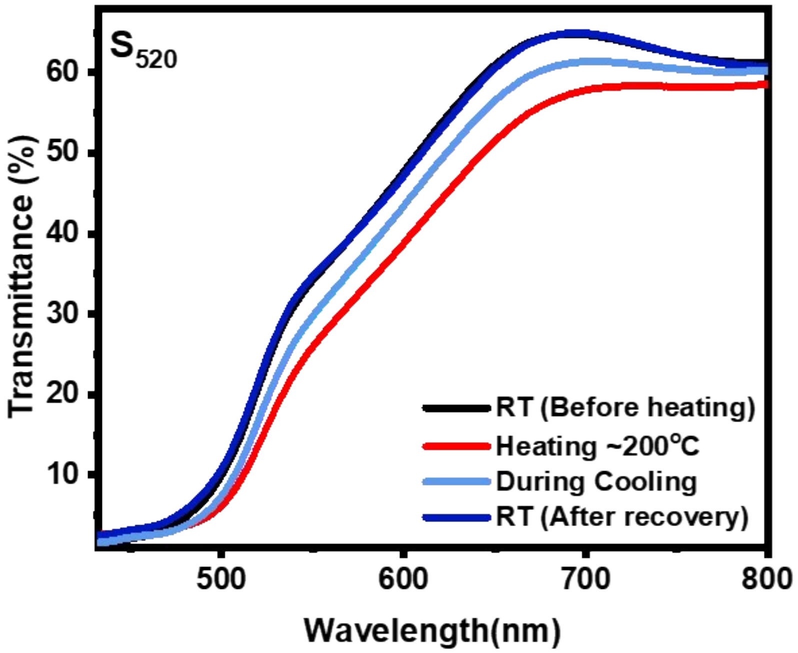

Figure S2: UV-spectra of the of V_2O_5 thin-films before and after heating at 200 ^oC
